# Analysis of Fluorescence Quenching of Coumarin Derivatives by 4-Hydroxy-TEMPO in Aqueous Solution

**DOI:** 10.1007/s10895-013-1342-3

**Published:** 2013-12-15

**Authors:** Krzysztof Żamojć, Wiesław Wiczk, Bartłomiej Zaborowski, Dagmara Jacewicz, Lech Chmurzyński

**Affiliations:** Faculty of Chemistry, University of Gdansk, Wita Stwosza 63, 80-952 Gdansk, Poland

**Keywords:** Coumarins, 4-hydroxy-TEMPO, Fluorescence quenching, Dynamic quenching

## Abstract

The fluorescence quenching of different coumarin derivatives (7-hydroxy-4-methylcoumarin, 5,7-dimethoxycoumarin, 7-amino-4-methyl-3-coumarinylacetic acid, 7-ethoxy-4-methylcoumarin, 7-methoxycoumarin, 7-hydroxycoumarin, 7-hydroxy-4-methyl-3-coumarinylacetic acid and 7-amino-4-methylcoumarin) by 4-hydroxy-TEMPO in aqueous solutions at the room temperature was studied with the use of UV–Vis absorption spectroscopy as well as a steady-state and time-resolved fluorescence spectroscopy. In order to understand the mechanism of quenching the absorption and fluorescence emission spectra of all coumarins along with fluorescence decays were recorded under the action of 4-hydroxy-TEMPO. The Stern-Volmer plots (both from time-averaged and time-resolved measurements) displayed no positive (upward) deviation from a linearity. The fluorescence quenching mechanism was found to be entirely dynamic, what was additionally confirmed by the registration of Stern-Volmer plots at different temperatures. The Stern-Volmer quenching constants and bimolecular quenching rate constants were obtained for all coumarins studied at the room temperature. The findings demonstrate the possibility of developing an analytical method for the quantitative determination of the free radicals’ scavenger, 4-hydroxy-TEMPO.

## Introduction

Coumarins comprise a very large class of phenolic substances. More than 1300 of them have been identified in nature, especially in green plants. The pharmacological and biochemical properties as well as therapeutic applications of simple coumarins depend on the pattern of substitution [1]. This class of bioactive compounds is known to act as free radicals’ scavengers [2], as well as possessing anticarcinogenic [3], antimicrobial [4], anti-inflammatory [5, 6], antiproliferative [7], antibacterial [8], anti-HIV [9] and antifungal [10] activities. Furthermore, many of coumarins are used as antioxidants [11, 12].

A lot of information about coumarins can be obtained by the studies on the fluorescence quenching [13–19], which refers to any process that decreases (or even eliminates) the fluorescence intensity or a quantum yield of luminescent species by interactions with other chemical compounds. The fluorescence quenching of organic molecules in solutions by different quenchers (for example aniline, bromobenzene, metal ions) has been widely studied by steady state and transient methods [20–24]. Although a large number of quenchers for coumarins have been identified, very few of them have been characterized in order to permit their use in studies performed in biological samples.

4-hydroxy-TEMPO is a stable membrane permeable nitroxide radical, which effectively protects cell and tissues from damages associated with oxidative stress conditions [25, 26]. In the present study we have used steady-state and time-resolved fluorescence measurements to investigate the quenching of fluorescence of eight coumarins by 4-hydroxy-TEMPO in aqueous solutions with a view to understand the nature of quenching mechanism involved in that system.

## Materials and Methods

The coumarins studied (7-hydroxy-4-methylcoumarin, 5,7-dimethoxycoumarin, 7-amino-4-methyl-3-coumarinylacetic acid, 7-ethoxy-4-methylcoumarin, 7-methoxycoumarin, 7-hydroxycoumarin, 7-hydroxy-4-methyl-3-coumarinylacetic acid, 7-amino-4-methylcoumarin) and 4-hydroxy-TEMPO were purchased from Sigma. Deionized water was used as a solvent. In order to avoid the self-quenching the solutions of all coumarins were prepared keeping the concentration fixed (1 · 10^-5^ M). The concentration of the stock solution of 4-hydroxy-TEMPO was constant too (0.25 M).

Absorption and emission spectra were recorded with the use of Perkin Elmer Lambda 650 UV–Vis spectrophotometer (the temperature - 25 °C, a scan speed – 266.75 nm · min^−1^, a slit - 2 nm, a data interval - 1 nm) and Cary Eclipse Varian spectrofluorimeter (the temperature - 25 °C, a scanning rate - 600 nm · min^−1^, excitation and emission slits - 5 nm, the averaging time - 0.1 s, a data interval - 1 nm). The excitation wavelength chosen for each coumarin was its absorption maximum. All spectra were recorded in the absence of 4-hydroxy-TEMPO and in its presence at different concentrations. The decreases in fluorescence intensities of all coumarins under the action of 4-hydroxy-TEMPO were observed at following temperatures: 15, 25, 35, 45 and 55 °C. In these experiments the fluorescence of each coumarin solution (1.95 mL, 1 · 10^−5^ M) was measured in the absence of 4-hydroxy-TEMPO and in the presence of different quencher’s concentrations (50 μL, 0.05–0.25 M). After each addition of 4-hydroxy-TEMPO the solution was gently stirred and the fluorescence intensity was measured.

Time-resolved fluorescence measurements were performed with the use of Edinburgh CD-900 spectrofluorimeter at the room temperature. In these experiments the fluorescence lifetimes of all coumarins studied (2.5 mL, 1 · 10^−5^ M) were measured using the single photon counting technique in the absence of 4-hydroxy-TEMPO and in the presence of its different amounts (20–100 μL, 0.25 M). The excitation wavelength was set to 340 nm in all the cases. After each addition of 4-hydroxy-TEMPO the solution was gently stirred and the fluorescence lifetime measured.

The molecular orbital package (A semiempirical molecular orbital program), MOPAC 2012 version 12.239 L (AM1 method) was used for the theoretical calculations of the ionization potentials and molecular radii of a series of coumarin derivatives. In addition the molecular radii were calculated after an optimization of geometry using Avogadro (version 1.1.0) Cross-Platform Computer Program for Building Molecules and Visualizing Structure and Analysis.

## Results and Discussion

**Fig. 1:**
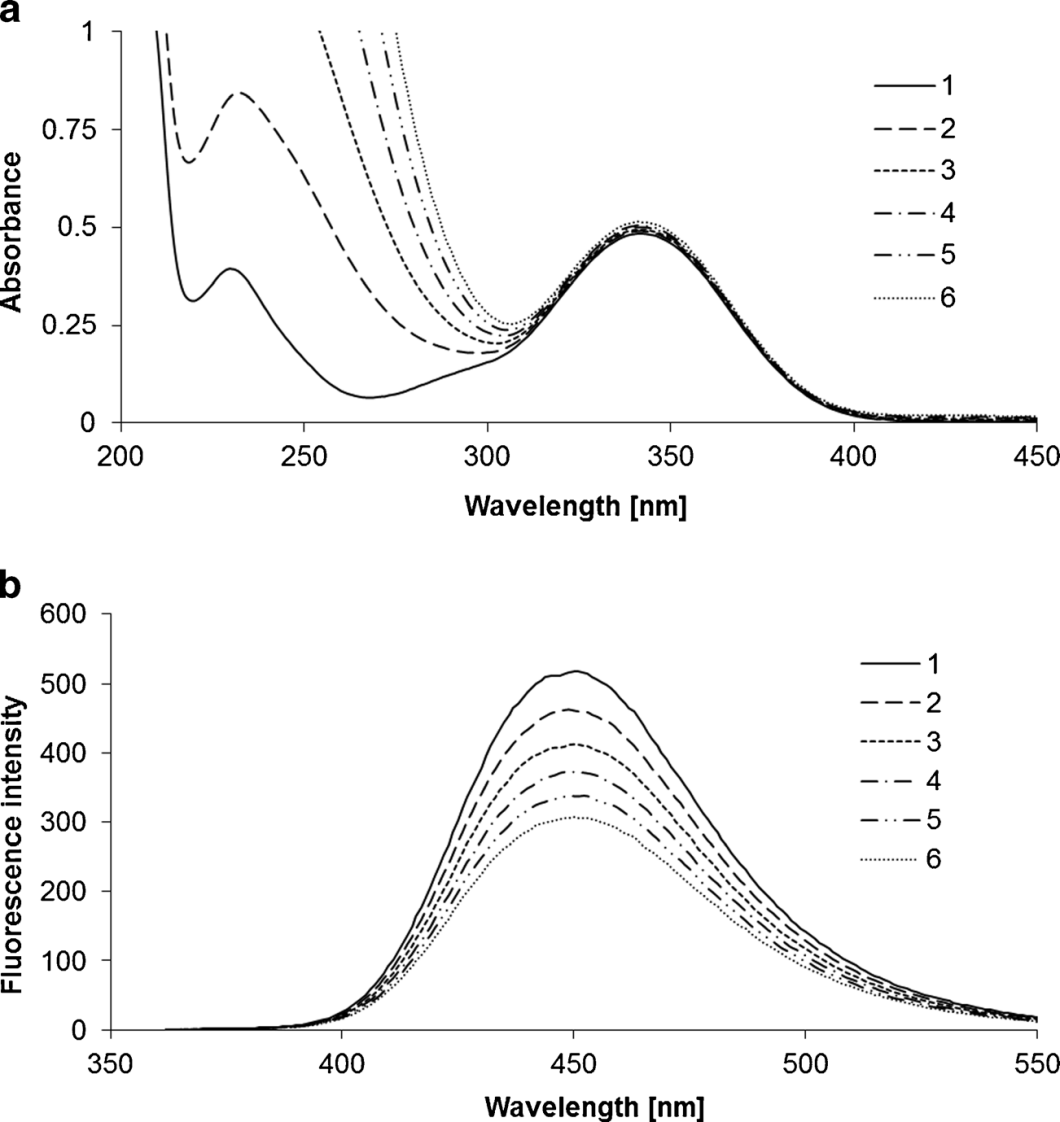
Absorption (**a**) and fluorescence emission (**b**) spectra of 7-amino-4-methyl-3-coumarinylacetic acid in the presence of increasing concentration of 4-hydroxy-TEMPO (spectrum 1 recorded in the absence of 4-hydroxy-TEMPO, spectrum 6 recorded in the presence of the highest concentration of 4-hydroxy-TEMPO)

UV absorption and fluorescence emission spectra of all coumarins studied in aqueous solutions were recorded in the absence of 4-hydroxy-TEMPO and in the presence of the increasing amounts of that radical. In all cases the same effect under the action of 4-hydroxy-TEMPO was observed. Appropriate spectra are presented on an example of 7-amino-4-methyl-3-coumarinylacetic acid in Fig. 1. From the registration of these spectra the following observations were made: (a) the fluorescence intensity of each fluorophore decreased with an increase of 4-hydroxy-TEMPO concentration; (b) the shape and band maxima of absorption and fluorescence spectra remained unchanged; (c) the intensity of fluorescence observed in the presence of 4-hydroxy-TEMPO was not time dependent (d) no other emission band of the fluorophore towards a longer wavelength was noticed. All these findings indicate that the permanent photochemistry is not involved in the quenching process and that the type of interactions between mentioned coumarins and 4-hydroxy-TEMPO is totally physical. Furthermore, they suggest that the quencher does not change the absorption and spectral properties of fluorophore and that the possibility of an emissive exciplex formation can be rejected.

**Fig. 2:**
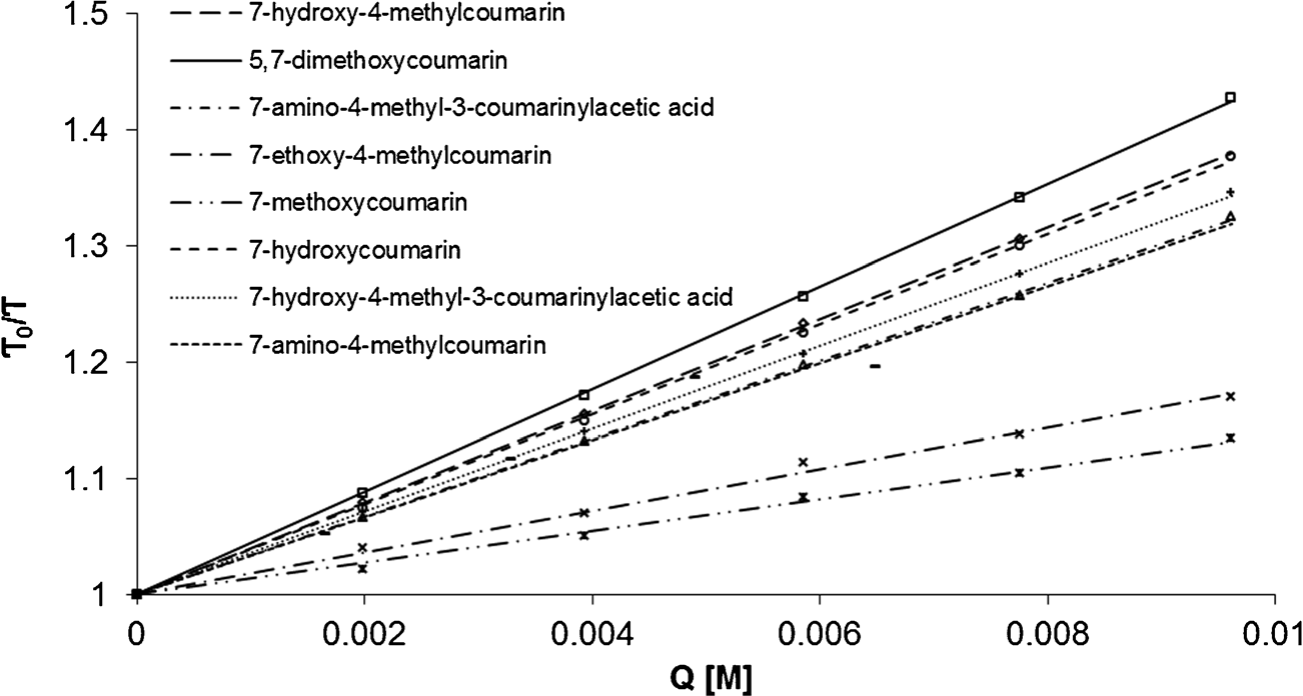
Stern-Volmer plots for the fluorescence quenching of coumarins studied by 4-hydroxy-TEMPO in aqueous solutions at the room temperature

To establish the mechanism of quenching for all coumarins studied the Stern-Volmer equation was analyzed with the use of a time-resolved fluorescence spectroscopy. In Fig. 2 there are shown the Stern-Volmer plots for the fluorescence quenching of coumarins by 4-hydroxy-TEMPO in aqueous solutions. The fluorescence lifetimes of each fluorophore in the absence and presence of 4-hydroxy-TEMPO were measured at different wavelengths, corresponding to the maximum of its emission (7-hydroxy-4-methylcoumarin: 451 nm; 5,7-dimethoxycoumarin: 453 nm; 7-amino-4-methyl-3-coumarinylacetic acid: 451 nm; 7-ethoxy-4-methylcoumarin: 388 nm; 7-methoxycoumarin: 395 nm; 7-hydroxycoumarin: 459 nm; 7-hydroxy-4-methyl-3-coumarinylacetic acid: 461 nm; 7-amino-4-methylcoumarin: 441 nm).

**Fig. 3:**
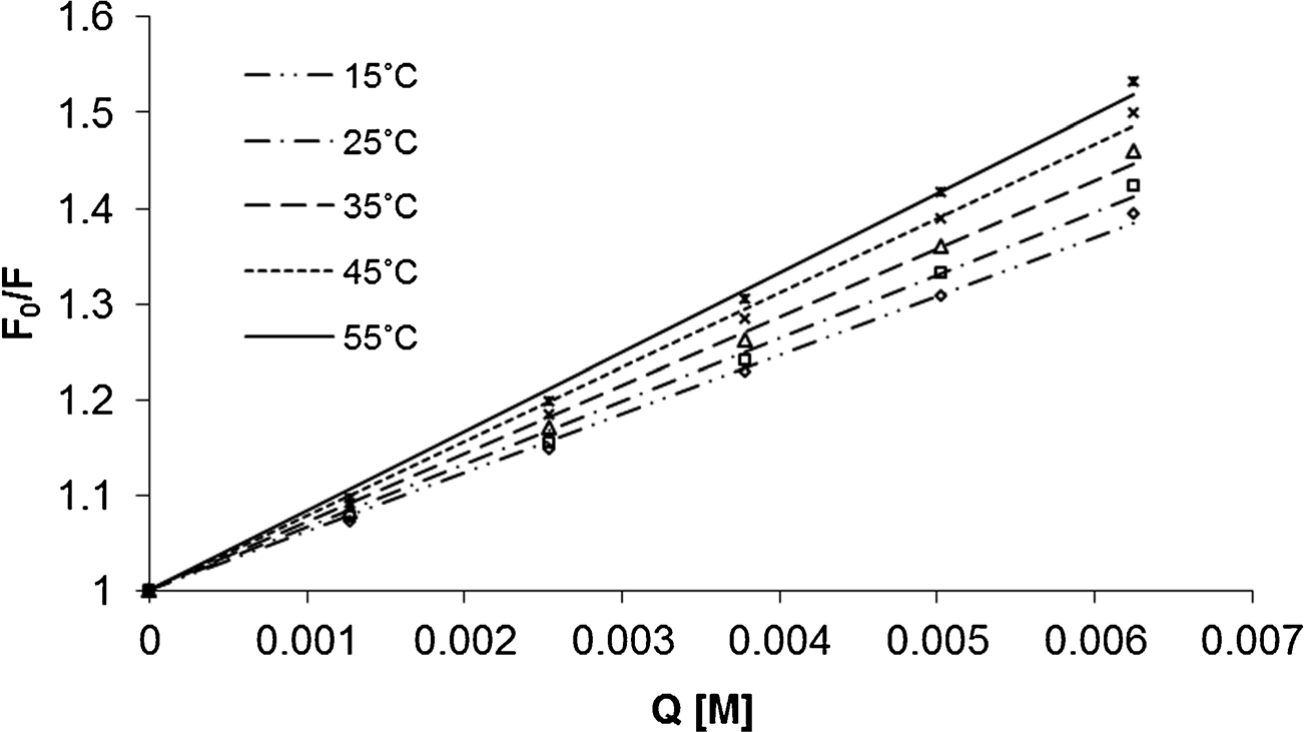
Stern-Volmer plots of the fluorescence quenching of the fluorescence of 7-amino-4-methyl-3-coumarinylacetic acid by 4-hydroxy-TEMPO in aqueous solutions at different temperatures

In the investigated range of quencher’s concentration the fluorescence quenching shows almost perfectly a linear dependence as the Stern-Volmer equation represents. There are no deviations from the linearity observed. It is a proof that there is no simultaneous presence of dynamic and static quenching. Furthermore, in case of all coumarins studied, the Stern-Volmer plot’s slope is higher than 0 (an addition of quencher has an influence on a fluorophore lifetime) what proves that the dynamic quenching mechanism occurs. In order to additionally confirm the dynamic mechanism of fluorescence quenching the effect of temperature on Stern-Volmer constants determined on the basis of steady-state measurements was established. Figure 3 shows the Stern-Volmer plots for the fluorescence quenching of 7-amino-4-methyl-3-coumarinylacetic acid by 4-hydroxy-TEMPO in aqueous solutions at five temperatures (15, 25, 35, 45, and 55 °C).

As it can be observed from Fig. 3 the Stern-Volmer plots’ slopes increase with an increase of temperature, what is characteristic for the dynamic quenching. For all coumarins studied the value of K_D_ obtained from steady-state measurements is approximately two times higher than the value of K_D_ obtained from time-resolved measurements. As 4-hydroxy-TEMPO absorbs light in the same wavelength as coumarins under study absorb and emit, it acts as a filter at the excitation and emission wavelength. According to that we decided to correct fluorescence emission intensities for inner filter effects. After the corrections discrepancies were decreased significantly and F_0_/F curves are in the range of experimental error to τ_0_/τ values (data not shown). In Table 1 the results of fluorescence quenching studied obtained for time-resolved measurements are collected. Fluorescence lifetimes measured are in a reasonable agreement with literature data [27–31].

**Table 1:** Stern-Volmer quenching constants (K_D_), linear correlation coefficients (r^2^), fluorescence lifetimes in the absence of quencher (τ_0_) and bimolecular quenching rate constants recovered for fluorescence quenching of coumarins by 4-hydroxy-TEMPO in aqueous solution

Coumarin	K_D_ [mol^−1^ · dm^3^]	*r* ^2^	τ_0_ [ns]	k_q_ [mol^−1^ · dm^3^ · s^−1^]
7-hydroxy-4-methylcoumarin	39.49	0.9999	5.66	6.98 · 10^9^
7-amino-4-methylcoumarin	33.22	0.9512	4.84	6.86 · 10^9^
7-hydroxy-4-methyl-3-coumarinylacetic acid	35.71	0.9999	5.14	6.95 · 10^9^
7-hydroxycoumarin	38.79	0.9996	5.36	7.24 · 10^9^
7-methoxycoumarin	13.69	0.9949	1.46	9.38 · 10^9^
7-ethoxy-4-methylcoumarin	17.97	0.9954	2.15	8.36 · 10^9^
7-amino-4-methyl-3-coumarinylacetic acid	33.55	0.9999	4.70	7.14 · 10^9^
5,7-dimethoxycoumarin	44.09	0.9998	7.14	6.18 · 10^9^

In order to understand better the mechanism of quenching ionization potentials of all coumarins studied as well as their molecular radii were determined with the use of theoretical calculations. They are gathered in Table 2.

**Table 2:** Ionization potentials (IP), molecular radii calculated with the use of Avogadro program (R_1_) and molecular radii calculated with the use of MOPAC (R_2_) recovered for fluorescence quenching of coumarins by 4-hydroxy-TEMPO in aqueous solution

Coumarin	IP [eV]	R_1_ [Å]	R_2_ [Å]
7-hydroxy-4-methylcoumarin	9.19	8.09	7.39
7-amino-4-methylcoumarin	8.70	8.12	7.78
7-hydroxy-4-methyl-3-coumarinylacetic acid	9.45	10.49	9.58
7-hydroxycoumarin	9.27	7.95	7.98
7-methoxycoumarin	9.18	9.27	9.37
7-ethoxy-4-methylcoumarin	9.10	10.61	10.17
7-amino-4-methyl-3-coumarinylacetic acid	8.93	10.92	9.72
5.7-dimethoxycoumarin	9.05	9.39	8.59

**Fig. 4:**
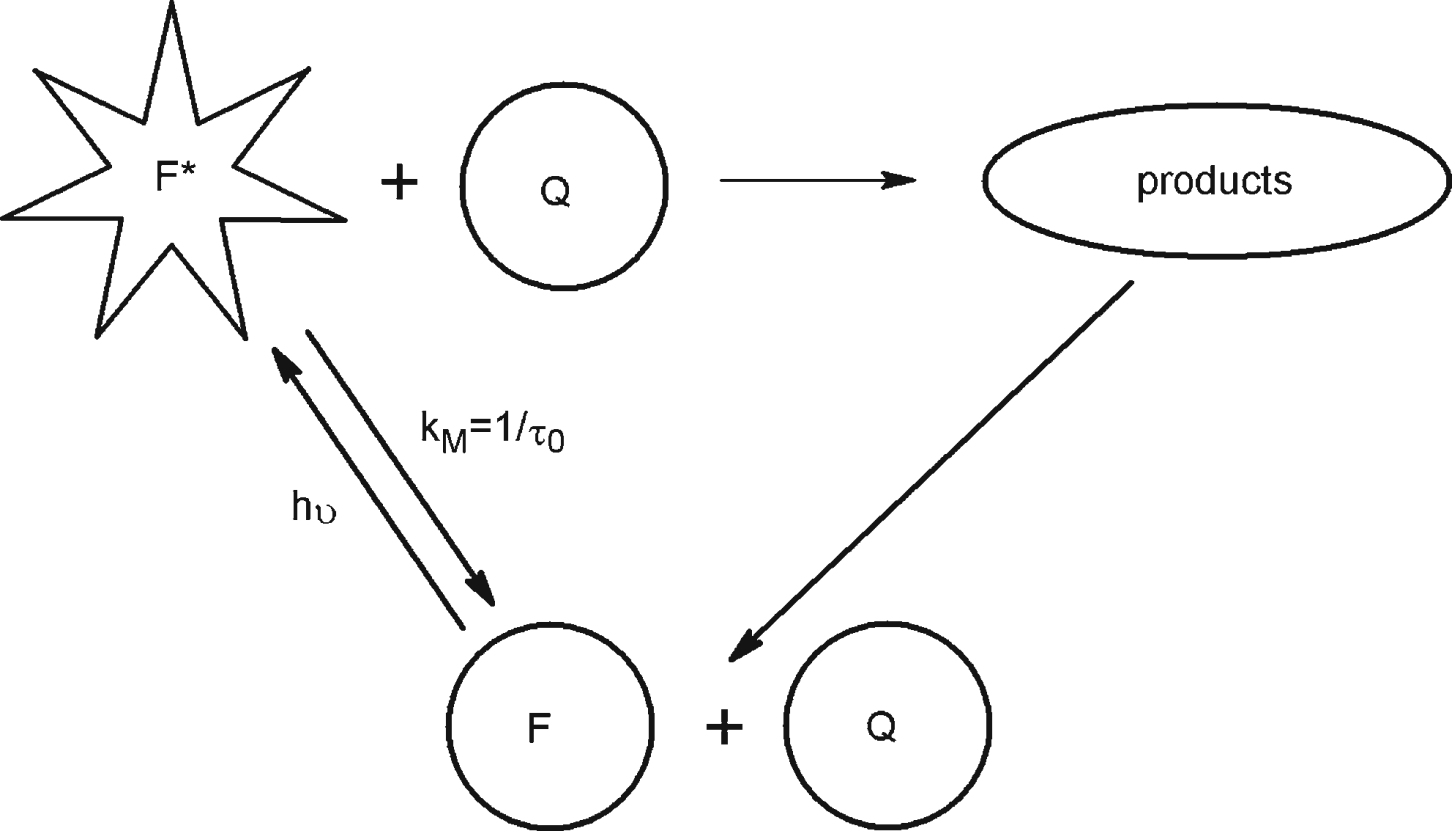
Schematic diagram of the mechanism of fluorescence quenching of coumarins studied by 4-hydroxy-TEMPO

As there is no correlation between bimolecular quenching rate constants and ionization potentials of coumarins studied the mechanism of electron transfer can be rejected. In addition there is also no correlation between bimolecular quenching rate constants and coumarins’ molecular radii. On the other hand, it may be explained by the fact that sizes of all coumarins studied are comparable and it is very difficult to register any correlation. Additionally, bimolecular quenching rate constants gathered in Table 1 are of the same order of magnitude as diffusion rate constants obtained for studies performed in water [32, 33]. On the basis of these results we can state that it is highly probable that the bimolecular process is diffusion-limited. The quenching mechanism can be additionally explained by the schematic diagram presented in Fig. 4.

## Conclusions

In this paper the fluorescence quenching of different coumarins by 4-hydroxy-TEMPO was investigated. The results show that the fluorescence of all coumarins studied is sensitive to the presence of 4-hydroxy-TEMPO and these compounds are quenched very effectively. There are no positive curvatures in Stern-Volmer plots at high quencher concentrations, indicating the influence of only one (dynamic) quenching mechanism. Performed steady-state and time-resolved measurements, as well as theoretical calculations let to assume that the bimolecular process is diffusion-limited. The biological significance of this work is proven by the fact that 4-hydroxy-TEMPO is used as the different radicals’ scavenger. Its detection and quantitative determination under physiological conditions might help to understand the mechanism of oxidative stress. As there are no significant differences in the fluorescence quenching of coumarins studied by 4-hydroxy-TEMPO it may be concluded that none of them is appropriate for quantitative measurements of scavenger’s amounts.
